# Female Breast Cancer Mortality Clusters in Shandong Province, China: A Spatial Analysis

**DOI:** 10.1038/s41598-017-00179-8

**Published:** 2017-03-07

**Authors:** Jie Chu, Chengchao Zhou, Xiaolei Guo, Jiandong Sun, Fuzhong Xue, Jiyu Zhang, Zilong Lu, Zhentao Fu, Aiqiang Xu

**Affiliations:** 1 The Department for Chronic and Non-communicable Disease Control and Prevention, Shandong Center for Disease Control and Prevention, Jinan, 250014 China; 2 School of Public Health, Shandong University, Jinan, 250012 China; 3 Institute for Resilient Regions, University of Southern Queensland, Springfield Central, Queensland, Australia; 4 School of Public Health and Social Work, Queensland University of Technology, Brisbane, Queensland Australia

## Abstract

This study aimed to detect the spatial distribution and high-risk clusters of female breast cancer mortality for the years 2011 to 2013 in Shandong Province, China. The urban-rural difference in the spatial distribution and clusters of disease mortality were also examined. Breast cancer mortality data were obtained from the Shandong Death Registration System (SDRS) during 2011 to 2013 and were adjusted for the underreporting rate. The purely spatial scan Statistics method was performed using Discrete Poisson model. Seven significant spatial clusters for high mortality of female breast cancer were detected in Shandong Province at the county level; these clusters were mainly located in the eastern, southern, southwestern, central and northern regions. The spatial distributions differed significantly between urban and rural populations. Population ageing influenced the distribution of breast cancer clusters for the urban eastern residents. This study provided evidence for the presence of clusters of breast cancer mortality in Shandong, China and found urban-rural difference in the clusters, which is helpful for developing effective strategies to control breast cancer in different areas.

## Introduction

Breast cancer is a leading cause of cancer death among women, with a steadily increasing incidence in many countries over the past 30 years^1, 2^. In 2012, approximately 1.7 million patients were diagnosed with breast cancer worldwide, and 0.5 million died of invasive breast cancer, accounting for 25.1% of total new cancer cases and 14.7% of cancer deaths^3^. Approximately half of breast cancer cases and 60% of deaths occurred in underdeveloped countries^3^.

Similar to other countries, breast cancer is the most common cancer in China, ranking first and sixth in new cases and cancer deaths among women in China, respectively^4^. Although the incidence and mortality of breast cancer among Chinese women were relatively lower than those in many other developed countries, a rapid increasing trend and an obvious regional difference have been observed in China since the 1990s, particularly in urban areas^5–9^. A report from four cities with complete tumor registration data showed that the crude incidence of female breast cancer has increased 2.23 to 3.77 times (a standardized incidence of 1.59 to 3.22 times) during 1988 to 2007^6^. According to the National Tumor Registration Report in China, the crude incidence of breast cancer in urban areas is 3 times greater (2.6 times after age standardization) than that in rural areas. Similarly, the mortality in urban areas is 47.83% higher (50.24% after age standardization) than that in rural areas^7^. The result of a national retrospective death survey showed that the mortality of female breast cancer in China has nearly doubled from the 1970s to 2004–2005, with a 36.10% increase in age-standardized mortality^8^. It is well documented that there are obvious geographic disparities in breast cancer, with higher mortalities in urban areas and eastern regions than those in rural areas and western regions^4, 9–11^. Shandong has one of the fastest-increasing incidences of breast cancer in China. Among the causes of cancer-related death, the rank of female breast cancer mortality has increased from the 8^th^ in the 1990s to the 5^th^ during 2004–2005^12, 13^.

Breast cancer is sometimes found concentrated in a specific region. The difference derived from such clusters exists not only between rural and urban areas but also among variant administrative units in urban or rural areas. When exploring the difference of cancer just by rural/urban status, we might overlook the potential clustering among different administrative units in rural or urban areas, which results in a failure to clearly depict real regional disparities^14^. Thus, some developed countries (i.e., the USA) have already attempted to monitor cancer-related data based on smaller units such as cities, counties, towns, and even villages^15–18^. Monitoring cancer-related data in smaller units can both provide detailed information about the incidence or mortality of the cancer and identify potential clusters more accurately^19^. The spatial statistical analysis developed by Kulldorff and Nagarwalla has been demonstrated to be an effective method for discerning geographical distribution and detecting spatial cluster based on smaller units^20, 21^.

Previous studies have demonstrated that there was a rural-urban difference in breast cancer; these studies were generally based on a sample from surveillance sites or a retrospective sampling survey of death causes in some specific regions of China. However, no studies have explored the clusters of breast cancer mortality based on an entire province, particularly a province with a large population of nearly 100 million (8% of China’s population). In addition, no studies, to the best of our knowledge, have examined the difference of spatial distributions between rural and urban areas. To remedy this situation, this study aimed to detect the geographic distribution and high risk areas of breast cancer mortality based on an entire population at the county level in Shandong, China and to examine the difference in spatial distribution between urban and rural areas.

## Methods

### Data collection

**Figure 1:**
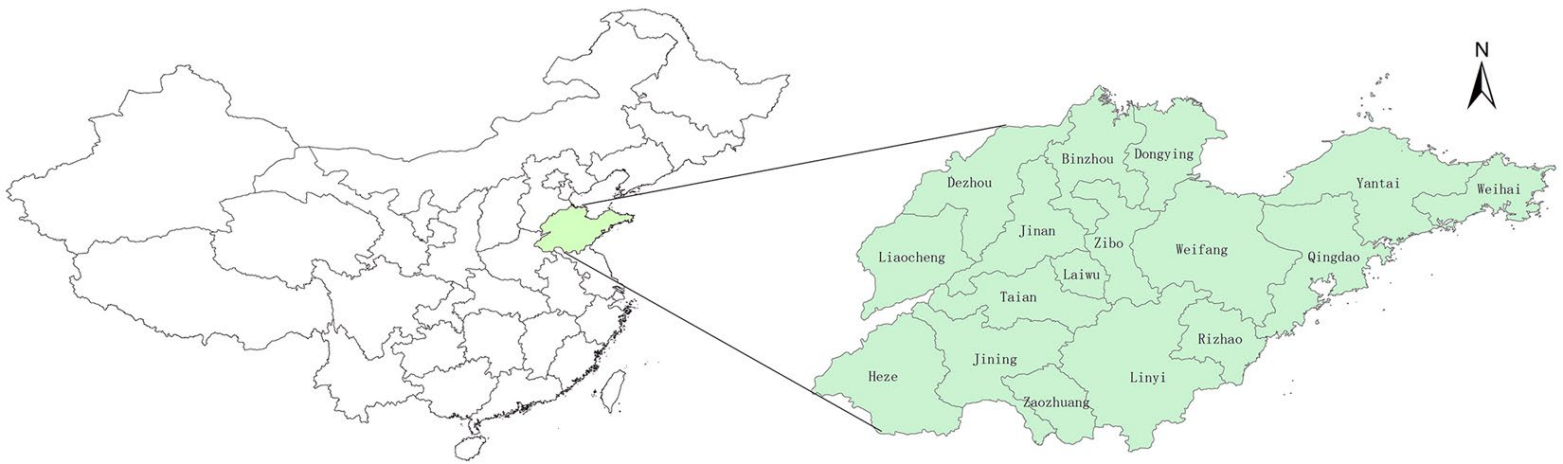
Location of Shandong Province, China (ArcGis 10.2 http://www.esri.com/software/arcgis/arcgisonline).

Shandong Province is located to the east of the mountain Tai-Hang and adjacent to the Bohai Sea and the Yellow Sea; it is the second-largest province, population-wise, in China (Fig. 1). Data on female breast cancer deaths have been collected through official surveillance of the Shandong Death Registration System (SDRS), which was established in 2006. The SDRS, which initially covered only the population of Disease Surveillance Points (DSPs), has collected data from the entire Shandong population since 2010. In this study, a case was defined as a death caused by malignant neoplasm of breast cancer (C50) according to the International Classification of Diseases, 10th Reversion (ICD-10), in the women residing in Shandong during 2011 to 2013^22^. These death data were adjusted based on the population-based underreporting investigation conducted in Shandong during 2011 to 2013 by the capture-mark-recapture (CMR) method^23^. CMR is an epidemiological method used to estimate the size of a targeted population with a specific characteristic^24^. It is hypothesized that M independent individuals in a randomly acquired sample from the targeted population with N individuals are marked and released to the original population. Then, another random sample is acquired with n independent individuals from the same targeted population to identify the number of marked individuals (m). An unbiased formula was used to estimate the size of the targeted population according to the two independent samplings. The population information was obtained from the Shandong statistical yearbook, and the age-specific population numbers for female permanent residents were acquired based on 142 counties/districts in Shandong during 2011 to 2013.

### Urban-rural classification

There are six levels in the Chinese administrative system: national, provincial, prefectural, county, township, and village level. In Shandong Province, there are 17 prefectures administrating 142 county-level units (counties/districts). Each county-level unit (county or district) usually includes two types of township level units: townships (in Chinese, we call “Xiangzhen”) and subdistricts (in Chinese, we call “Jiedao”). The former consists of a town centre and dozens of surrounding villages. The latter is comprised of urban communities or suburbs. We defined the rural population as people living in townships, and the urban population as people living in subdistricts. This classification is virtually identical to the recommended methods by the National Statistical Bureau, which were used in the recent census^25^. There are no rural townships in 17 urban districts and thus only urban population was defined in such districts. For 5 counties with predominantly rural population, the data of subdistricts population were unavailable and we treated the population of the entire counties as rural population. All in all, we defined 262 sub-county level units for assessment totally. Among them, 137 were urban units and 125 were rural units. Of which, 5 units with small populations, including 4 urban units and 1 rural unit, had no breast cancer deaths during the study period, and we treated their mortalities as zero.

### Statistical analysis

To alleviate mortality variations in small populations and areas, the average reported mortality rate (ARMR) of breast cancer was calculated in each county/district as a ratio of total deaths over the corresponding population from 2011 to 2013. The ARMR of breast cancer was displayed by the GIS-based maps at the county level to visualize the distribution patterns of breast cancer and the high-risk (hotspot) areas in Shandong Province. The county-level point layer, containing information on latitudes and longitudes of the central points of each county, was created by GIS with a ratio of 1:100,000 to draw the maps.

We performed purely spatial analysis using a Discrete Poisson model to detect the spatial distribution of female breast cancer deaths in Shandong Province. Age and urban/rural status were adjusted to discern their effects on breast cancer clusters. Statistical significance of clustering was based on the Monte Carlo hypothesis testing^26^ by comparing the likelihood ratio test statistic from the observed data set with the test statistic from 999 random data sets generated under the null hypothesis of no clustering. The level of statistical significance was set as 0.05 in this study. The spatial scan statistics detected the disease clusters by gradually scanning a window across space and comparing the number of observed and expected cases inside the window. In our study, we specified the maximum spatial cluster size as one with 50% of the population at risk and conducted the scanning window in the shape of a circle. The most likely cluster was defined as that with the maximum likelihood ratio (LLR) with statistical significance. Secondary cluster were also reported that rejected the null hypothesis but did not overlap with the most likely cluster.

The calculation of the ARMR at the county level was conducted by Stata Version 12.0 (Stata Cor., College Station, TX, USA). The county-level polygon maps at the 1:100,000 scale of the ARMR and disease cluster were drawn using the software ArcGis 10.2 (ESRI Inc., Redlands, CA, USA)^27^. A spatial scan statistics analysis was performed to examine the presence of female breast cancer clusters using SaTScan v9.1.1^28^ developed by the National Cancer Institute (NCI, Boston, MA, USA).

### Study approval

The entire research protocol of this study was approved by the Ethics Committee of Preventive Medicine in Shandong Center for Disease Control and Prevention in 2013, with permission No. of 2013020. All methods such as data collection and analysis method were performed in accordance with the relevant guidelines and regulations.

## Results

### Descriptive analysis

In all, 11,510 breast cancer deaths occurred in the Shandong female population during the years 2011 to 2013, with an ARMR of 8.16 per 100,000 women. Of these, 11,505 women (99.96%) had complete information including their place of residence. For urban and rural units, the ARMR was 8.85 and 7.80 per 100,000 women, respectively. The ARMR at the county level ranged from 0.57 to 17.95 per 100,000 women.

**Figure 2:**
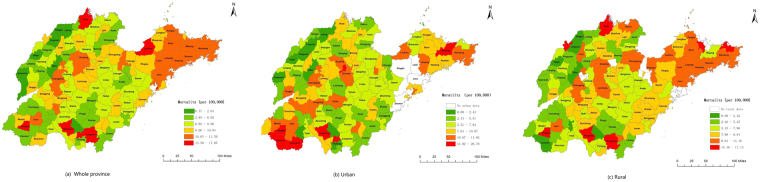
Geographic distribution of female breast cancer mortality at the county level in Shandong on Urban/Rural Status for the years 2011 to 2013. Note: In Fig. 2b, 5 county-level units (counties) with predominantly rural population were regarded as completely rural units and had no urban data because of unavailable population data of subdistricts; In Fig. 2c, 17 county-level units (districts) had no rural townships and thus only urban populations were defined in these districts. (ArcGis 10.2 http://www.esri.com/software/arcgis/arcgisonline).

Figure 2a shows the geographic distribution of female breast cancer mortality in Shandong Province, in which green represents lower mortality than expected and red represents higher mortality than expected. In general, female breast cancer mortality displayed a decreasing trend from the eastern region to the western region, but some counties of the western region were still at a high risk of mortality. High mortality rates were mainly concentrated in the eastern region and some counties of the southwestern region in Shandong, including most of the counties in Yantai, Qingdao and Weihai cities; Dingtao county and Juye county in Heze city; Tengzhou county and Shizhong district in Zaozhuang city; and Cangshan county in Linyi city. Lower mortality rates were mainly located in the western and northern areas of Shandong.

For urban units, the mortalities of the eastern and southwestern regions were relatively higher than those of the other areas. The highest mortality rates were mainly located in the southwestern region of Shandong, including Dingtao county, Cao county and Shan county in Heze city, and Tengzhou county and Shizhong district in Linyin city (Fig. 2b). For rural units, the regions with the highest mortality presented a dispersed distribution and were mainly located in the surrounding areas in Shandong, such as Fushan district in Yantai city, Wudi county in Binzhou city, Decheng district in Dezhou city, Dingtao county and Juye county in Heze city, Tengzhou county in Zaozhuang city, and Cangshan county in Linyi city, but the mortality of female breast cancer gradually decreased from the eastern region to the western region (Fig. 2c).

### Spatial scan statistics analysis

**Figure 3:**
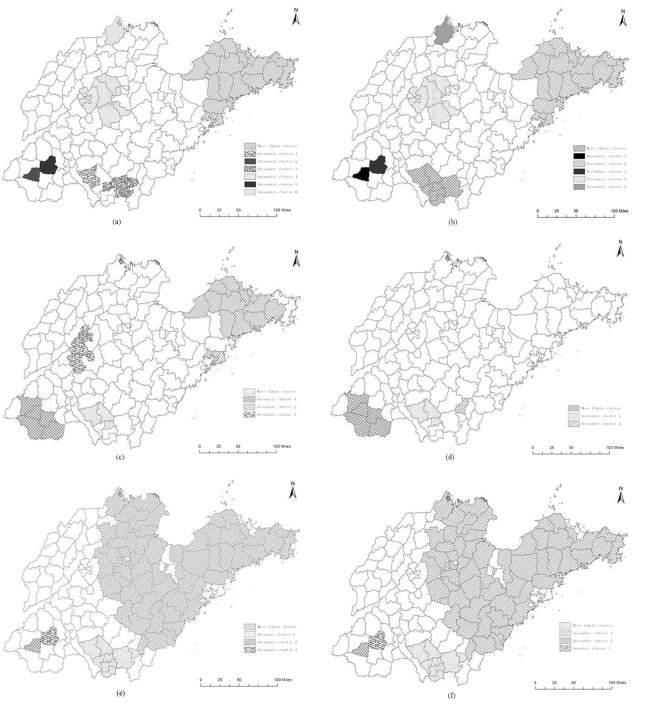
Female breast cancer mortality clusters at the county level in Shandong for the years 2011 to 2013 using the spatial scan statistical analysis (**a**) Total without adjustment; (**b**) Total adjusted for age; (**c**) Urban without adjustment; (**d**) Urban adjusted for age; (**e**) Rural without adjustment; (**f**) Rural adjusted for age (ArcGis 10.2 http://www.esri.com/software/arcgis/arcgisonline).

The spatial scan statistics analysis detected seven significant spatial clustering areas of breast cancer mortality in the entire province (Table 1 and Fig. 3a), suggesting that breast cancer mortality was not randomly distributed. The most likely cluster was located in the eastern region of Shandong Province, namely in Jiaodong Peninsula, including 25 counties with a relative risk (RR) of 1.46 compared to the rest of Shandong Province. The 1st and 3rd secondary clusters were located in the southern region, including 4 counties (Tengzhou county and Shizhong district in Zaozhuang city and Luozhuang district and Cangshan county in Linyi city) with RRs of 2.01 and 1.54, respectively, compared to the other areas. The 2nd and 5th secondary clusters were located in the southwestern region, including 2 counties in Heze city (Dingtao and Juye counties) with RRs of 2.24 and 1.56, respectively. The 4th secondary cluster was located in the central region of Shandong, including 9 counties/districts (Shizhong district, Lixia district, Licheng district, Tianqiao district and Zhangqiu county in Jinan city; Boshan district and Zhoucun district in Zibo city; Laicheng district in Laiwu city; and Zouping county in Binzhou city) with a RR of 1.31. The remaining secondary cluster was located in the northern region and only included one county (Wudi county in Binzhou city) with a RR of 1.59. After controlling for age, six clusters were detected in which the spatial distribution was consistent with that before controlling for age (Table 1 and Fig. 3b).

**Table 1:** Results of the spatial scan analysis of female breast cancer mortality at the county level in Shandong for the years 2011 to 2013.

Cluster	County amount	Cases	Expected	RR	LLR	P value
***Total without adjustment for age***						
Most likely cluster	25	2265	1651	1.46	125.69	<0.001
Secondary cluster 1	1	399	196	2.01	78.17	<0.001
Secondary cluster 2	1	174	79	2.24	46.31	<0.001
Secondary cluster 3	3	443	287	1.54	36.65	<0.001
Secondary cluster 4	9	942	751	1.31	30.47	<0.001
Secondary cluster 5	1	197	124	1.56	17.15	<0.001
Secondary cluster 6	1	90	55	1.59	8.29	<0.01
***Total adjusted for age***						
Most likely cluster	7	668	420	1.64	65.94	<0.001
Secondary cluster 1	1	133	54	2.48	40.97	<0.001
Secondary cluster 2	24	1315	1088	1.25	25.95	<0.001
Secondary cluster 3	1	154	85	1.82	22.47	<0.001
Secondary cluster 4	9	633	516	1.25	13.19	<0.001
Secondary cluster 5	1	66	38	1.77	8.86	<0.05
***Urban without adjustment for age***						
Most likely cluster	3	211	94	2.32	55.61	<0.001
Secondary cluster 1	5	287	171	1.73	34.21	<0.001
Secondary cluster 2	19	871	706	1.29	21.74	<0.001
Secondary cluster 3	7	489	396	1.27	11.31	<0.01
***Urban adjusted for age***						
Most likely cluster	5	233	119	2.04	44.77	<0.001
Secondary cluster 1	3	153	65	2.42	43.99	<0.001
Secondary cluster 2	1	86	50	1.73	10.69	<0.01
***Rural without adjustment for age***						
Most likely cluster	59	3789	3159	1.42	110.66	<0.001
Secondary cluster 1	8	692	467	1.53	51.16	<0.001
Secondary cluster 2	1	124	61	2.05	25.28	<0.001
Secondary cluster 3	1	162	100	1.63	16.2	<0.001
***Rural adjusted for age***						
Most likely cluster	8	504	318	1.65	49.7	<0.001
Secondary cluster 1	59	2453	2155	1.28	36.24	<0.001
Secondary cluster 2	1	93	42	2.26	23.69	<0.001
Secondary cluster 3	1	127	68	1.88	20.28	<0.001

Note: “Most likely cluster” was defined when the maximum log likelihood ratio (LLR) with statistical significance was detected by Monte Carlo simulation in the spatial analysis; the other LLR values with statistical significance were identified as the “Secondary cluster”. The relative risk (RR) was the ratio of the mortality inside a cluster area to the mortality outside a cluster area.

The result of the spatial scan analysis showed that there were four detected clusters of breast cancer mortality in the urban areas of Shandong Province (Table 1 and Fig. 3c). The most likely cluster was located in the southern region and included 3 counties with a RR of 2.32 compared to the rest of Shandong. The other clusters were located in the southwestern, eastern and central regions of Shandong with RRs of 1.73, 1.29 and 1.27, respectively, compared to the remaining areas. After controlling for age, only three clusters were detected and located in the southwestern and southern regions (Table 1 and Fig. 3d). The most likely cluster was located in the southwestern region, with a RR of 2.04. The two secondary clusters were both located in the southern region, with RRs of 2.42 and 1.73, respectively.

Four clusters were detected in the rural areas of Shandong using a purely spatial scan statistical analysis (Table 1 and Fig. 3e). The most likely cluster was located in Shantung Peninsula and included 59 counties with a RR of 1.42. The other secondary clusters were located in the southern and southwestern regions with RRs of 1.53, 2.05 and 1.63, respectively, compared to the other areas of Shandong. After controlling for age, the spatial distribution of the clusters was consistent with that before controlling for age (Table 1 and Fig. 3f).

## Discussion

This study detected, for the first time, the spatial distribution and high-risk clusters of female breast cancer mortality at the county level in an entire population of Shandong Province in China. Consistent with previous studies, the current study also found significantly higher mortality in the eastern region than that in the western region. This difference remained significant even when the mortality was standardized by age (data not presented). Jing Han and his colleague’s study^29^ showed significant regional differences in female breast cancer mortality at the county level in Shandong, with the highest county (9.99 per 100,000) having nearly four times as high a rate as the lowest county (2.63 per 100,000). Another study in Shandong^30^ showed that female breast cancer mortality was higher in the eastern region than that in the western region. The study also found that the mortality was higher in urban areas than that in rural areas.

The spatial scan analysis in this study detected seven significant clusters of breast cancer mortality, which were mainly located in the eastern, southern, southwestern, central and northern areas of Shandong. These clusters remained significant after controlling for age, which indicated that the influence of population ageing on the clusters of breast cancer mortality was not significant.

Urban-rural difference in breast cancer has been demonstrated both in China^11, 30^ and in other countries^31–33^. In the current study, this difference was also examined. Two major populations (urban and rural residents) were classified based on sub-county level units (townships/subdistricts) to respectively explore the spatial distributions and clusters of breast cancer mortality in urban and rural areas. The results indicated that the geographical distribution of breast cancer mortality in urban areas was not completely accordant with that in rural areas, even though a gradual increase in mortality from western to eastern regions was observed both in urban and rural areas. Further study is necessary to identify the clusters of breast cancer mortality in urban and rural areas respectively.

In urban areas, four significant spatial clusters were detected by the spatial scan analysis; these were located in the eastern, southern, southwestern and central regions. After controlling for age, the clusters in the southwestern and southern regions were still discerned, whereas the clusters located in the eastern and central region disappeared and a new cluster including only one county appeared in the southern region. This indicated that the clusters in the eastern and central regions might result from the regional population ageing. Three clusters were found in rural areas located in the eastern, southern and southwestern regions. When controlling for age, the results remained the same, which suggested that population ageing did not affect the clusters in rural areas.

Similar to previous studies, female breast cancer mortality in this study was higher in the eastern region than that in the western region^13, 30^. Some previous studies have reported a positive correlation between the socioeconomic level and breast cancer mortality^34, 35^. In Shandong, the economy in the eastern region is more developed compared to the central and western regions. This regional difference in breast cancer mortality might be a result of the imbalance in the development of the regional economy in Shandong Province^8, 10, 34^.

There was a difference in the spatial distribution and clusters of breast cancer between urban and rural areas in this study; this difference has not been reported in previous studies. The clusters were observed in eastern rural areas regardless of adjustment for age. However, no clusters were observed in eastern urban areas when adjusted for age. In the eastern urban areas, accessibility to high quality health services was easier for women diagnosed with breast cancer than for women in rural areas. In addition, the urban women tended to receive physical examinations more frequently, which is helpful for detecting breast cancer in its early stage^36^. Thus, the women in eastern urban areas would likely have higher survival rates after the diagnosis of breast cancer than rural women.

The results in our study suggested that there were also some small clusters in the underdeveloped western and southwestern regions in Shandong. Women living in these areas may have lower survival rates because of delayed detection and poor access to high quality health services^37^. It is also likely that cancer-related determinants existed to lead to the high incidence of breast cancer in these areas. Moreover, the inherent reluctance due to cultural barriers and cancer fatalism in Chinese women may also hamper screening and treatment efforts, particularly in older women and those from groups with low socioeconomic status^38–40^.

This study has several limitations. First, although the death data were adjusted for underreporting rates, we could not exclude the possibility that the underreporting rates vary across counties/districts and that some counties/districts or towns/subdistricts might have more missing cases for various reasons. In addition, given the delay of population statistical data collection, we could not obtain official data from the population statistical bureau at the beginning of our study, and thus the total numbers of the population from 2010 to 2012, instead of from 2011 to 2013, were used. Second, we only analysed the spatial distribution of breast cancer mortality in the entire province during a short period (the average mortality was calculated from 2011 to 2013) using a purely spatial analysis. Further studies are necessary to evaluate the spatial and temporal changes in the distribution of breast cancer mortality using longitudinal data from a longer surveillance period. Third, we did not assess potential influencing factors associated with clustering. Although we had hoped to obtain socioeconomic and environmental information from the official surveillance data, we were unable to do so. Future research is necessary to identify potential associated factors in the clusters.

## Conclusion

In conclusion, this study detected the spatial distribution and clusters of female breast cancer mortality in Shandong Province, China. The results demonstrated that the mortality was higher in the eastern regions than in the western regions; an urban-rural difference in the clusters was also identified. The study indicated that the spatial distribution and clusters of breast cancer mortality differed between urban and rural areas. The identified clusters in eastern rural areas were not observed in eastern urban areas. This study may provide public health officials with necessary information about statistically significant clusters of breast cancer mortality in urban and rural regions and thus enable them to perform more effective and targeted strategies to control breast cancer in different areas. More detailed individual-level investigations are necessary in the identified clusters to evaluate potential determinants for female breast cancer mortality.
